# Replication of the coronavirus genome: A paradox among positive-strand RNA viruses

**DOI:** 10.1016/j.jbc.2022.101923

**Published:** 2022-04-10

**Authors:** Emeline Grellet, India L'Hôte, Adeline Goulet, Isabelle Imbert

**Affiliations:** Aix Marseille Université, Centre National de la Recherche Scientifique, AMU CNRS UMR 7255, LISM, Marseille, France

**Keywords:** +RNA virus, coronavirus, RNA-dependent RNA polymerase, RNA replication and proofreading, macromolecular complexes, CoV, coronavirus, EAV, equine arteritis virus, ExoN, exoribonuclease, GTase, guanylyltransferase, MERS-CoV, Middle East respiratory syndrome-CoV, MHV, murine hepatitis virus, MTase, methyltransferase, nsp, nonstructural protein, PRF, programmed -1 ribosomal frameshifting, RdRp, RNA-dependent RNA polymerase, RTCs, replication/transcription complexes, SARS-CoV, severe acute respiratory syndrome-CoV, sg mRNAs, subgenomic messenger RNAs, TRS, transcriptional regulatory sequence, UTR, untranslated region, ZBD, zinc-binding domain

## Abstract

Coronavirus (CoV) genomes consist of positive-sense single-stranded RNA and are among the largest viral RNAs known to date (∼30 kb). As a result, CoVs deploy sophisticated mechanisms to replicate these extraordinarily large genomes as well as to transcribe subgenomic messenger RNAs. Since 2003, with the emergence of three highly pathogenic CoVs (SARS-CoV, MERS-CoV, and SARS-CoV-2), significant progress has been made in the molecular characterization of the viral proteins and key mechanisms involved in CoV RNA genome replication. For example, to allow for the maintenance and integrity of their large RNA genomes, CoVs have acquired RNA proofreading 3′-5′ exoribonuclease activity (in nonstructural protein nsp14). In order to replicate the large genome, the viral-RNA–dependent RNA polymerase (RdRp; in nsp12) is supplemented by a processivity factor (made of the viral complex nsp7/nsp8), making it the fastest known RdRp. Lastly, a viral structural protein, the nucleocapsid (N) protein, which is primarily involved in genome encapsidation, is required for efficient viral replication and transcription. Therefore, CoVs are a paradox among positive-strand RNA viruses in the sense that they use both a processivity factor and have proofreading activity reminiscent of DNA organisms in addition to structural proteins that mediate efficient RNA synthesis, commonly used by negative-strand RNA viruses. In this review, we present a historical perspective of these unsuspected discoveries and detail the current knowledge on the core replicative machinery deployed by CoVs.

Over the last 100 years, a large proportion of viral outbreaks have been caused by RNA viruses (*e.g.*, influenza, dengue, Chikungunya, Zika, Ebola viruses, etc.). Indeed, in contrast to DNA organisms, RNA-dependent RNA polymerases (RdRps) that ensure viral RNA genome synthesis are devoid of coreplicative and postreplicative fidelity-enhancing pathways (1). Thus, a high mutation frequency during viral RNA genome replication results in the RNA virus quasispecies concept, which is defined as complex distributions of closely related variant genomes (for reviews, see (2, 3)). These closely related viral genomes are mostly responsible for viral genome diversity and, consequently, facilitate the emergence or rapid adaptation of RNA viruses. Moreover, the high mutation rate of RdRps is thought to have constrained RNA virus genome sizes (<15 kilobases).

Among eukaryote RNA viruses, those with positive polarity genomes (+RNA) can be directly used by host ribosomes to produce viral proteins. By convention, they are divided into structural proteins and proteins for viral genome amplification (so-called nonstructural proteins (nsps)). For the latter, in addition to the key RdRp activity already mentioned, some other essential enzymatic activities are commonly found in +RNA viruses, such as protease and RNA helicase. Besides, depending on the virus family, less common or unique activities can be found. In any case, cytoplasmic viral nsps assemble in protein complexes, which, in association with modified intracellular membranes, form viral replication organelles. They offer a favorable microenvironment for the amplification of the viral genome through a full-length minus-strand intermediate, serving in turn as a template for new viral messenger RNA genome production. In addition, these viral organelles may shield viral dsRNA intermediates from innate immune sensors. Neosynthesized +RNA genomes can then either be translated into additional viral proteins, serve as a template for additional minus-strand RNA synthesis, or be packaged into progeny virions.

## The coronaviruses

Before 2003, coronaviruses (CoVs) were mainly a veterinary problem with substantial economic losses (for instance, the avian infectious CoV or the porcine epidemic diarrhea virus), whereas endemic human CoV strains (such HCoV-229E and OC43) were considered benign to human health. They typically caused mild upper respiratory tract diseases or the common cold. It was only at the beginning of the 21st century that CoVs became a real threat to mankind, with the emergence of severe acute respiratory syndrome-CoV (SARS-CoV) in December 2002 (4), Middle East respiratory syndrome-CoV (MERS-CoV) in 2012 (5, 6), and now of SARS-CoV-2 (7). CoVs are presently divided into four clades, named alpha-, beta-, gamma-, and delta-CoVs, and include viruses known to infect humans, bats, other mammals, and several avian species. The three highly pathogenic CoVs to humans are part of the beta-CoV clade. CoVs together with toroviruses form the *Coronaviridae* family, which together with the *Arteriviridae*, *Roniviridae*, and *Mesoniviridae* families belongs to the *Nidovirales* order (8). In addition, in 2018, a novel unclassified nidovirus named planarian secretory cell nidovirus was characterized and extended the nidovirus genome size, harboring a genome of 41.1 kb (9). Moreover, viral metagenomics have greatly expanded the *Nidovirales* tree with 88 formally recognized virus species, resulting in ongoing discussions to revise the taxonomy classification of the order (10, 11, 12).

**Figure 1:**
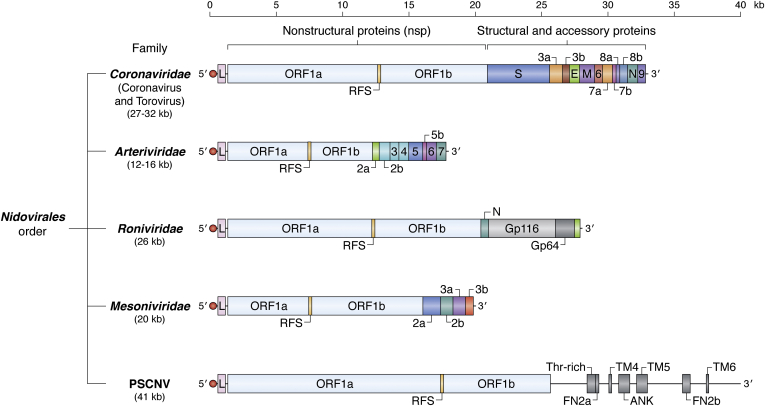
**Genomic and proteomic organization of the *Coronaviridae*, *Arteviridae*, *Roniviridae*, and *Mesoniviridae* families, as well as the PSCNV forming the *Nidovirales* order.** PSCNV, the planarian secretory cell nidovirus; the *red ball* represents the cap structure and the *yellow box* indicates the ribosome frameshifting signal (RFS). The structural and accessory proteins were colored depending on their functions. ANK, ankyrin domain; E, envelop; FN2a/b, fibronectin type 2 domains; M, membrane, N; nucleoprotein; S, spike; thr-rich, region enriched with Thr residue; TM, transmembrane domain.

Nidoviruses are positive-sense single-stranded RNA viruses, comprising the largest viral RNAs known to date (from 12.7 to 41.1 kb) (9). The emergence of the largest nidoviruses (>20 kb) is associated, in part, with the acquisition of a viral proofreading exoribonuclease (ExoN), which will be discussed later (4, 13, 14). With the exception of planarian secretory cell nidovirus, all these viruses exhibit a similar genome organization, with two large overlapping nsp ORFs (ORF1a and ORF1b) encoded in the 5′ two-thirds of the genome in addition to multiple structural and accessory protein ORFs encoded in the 3′ third (Fig. 1). The latter are translated from a nested set of subgenomic messenger RNAs (sg mRNAs), which is one of the nidovirus-specific hallmarks (*L. nidus* = nest). sg mRNAs are expressed from a complex mechanism involving discontinuous RNA synthesis. Remarkably, these sg mRNAs harbor common 5′- and 3′-terminal sequences with the full-length genome.

In this review, we relay the history of the discoveries and detail the current knowledge on the core replicative machinery deployed by CoVs to replicate, transcribe, and maintain the integrity of their extraordinarily large genome (∼30 kb for the three highly human pathogenic CoVs). Whenever possible, a parallel is made with arteriviruses, the short vertebrate nidoviruses that share the same genomic organization as well as sg mRNAs transcription, but without ExoN proofreading activity.

## Overview of CoV RNA genome translation and replication

The polycistronic RNA genome of CoVs is flanked by two untranslated regions (UTR) at its 5′ and 3′ ends. The 5′UTR, of about 350 nucleotides (nt), contains a 5′ cap structure followed by the leader and a transcriptional regulatory sequence (TRS), both involved in sg mRNA production (discussed later). The CoV 3′UTR consists of 300 to 500 nucleotides plus a poly(A) tail of approximately ∼47 nt on average (15). Both genomic ends fold into secondary and higher-order structures, functionally important for RNA–RNA interactions and for the binding of viral and cellular proteins during its translation, replication, and transcription processes (16).

**Figure 2:**
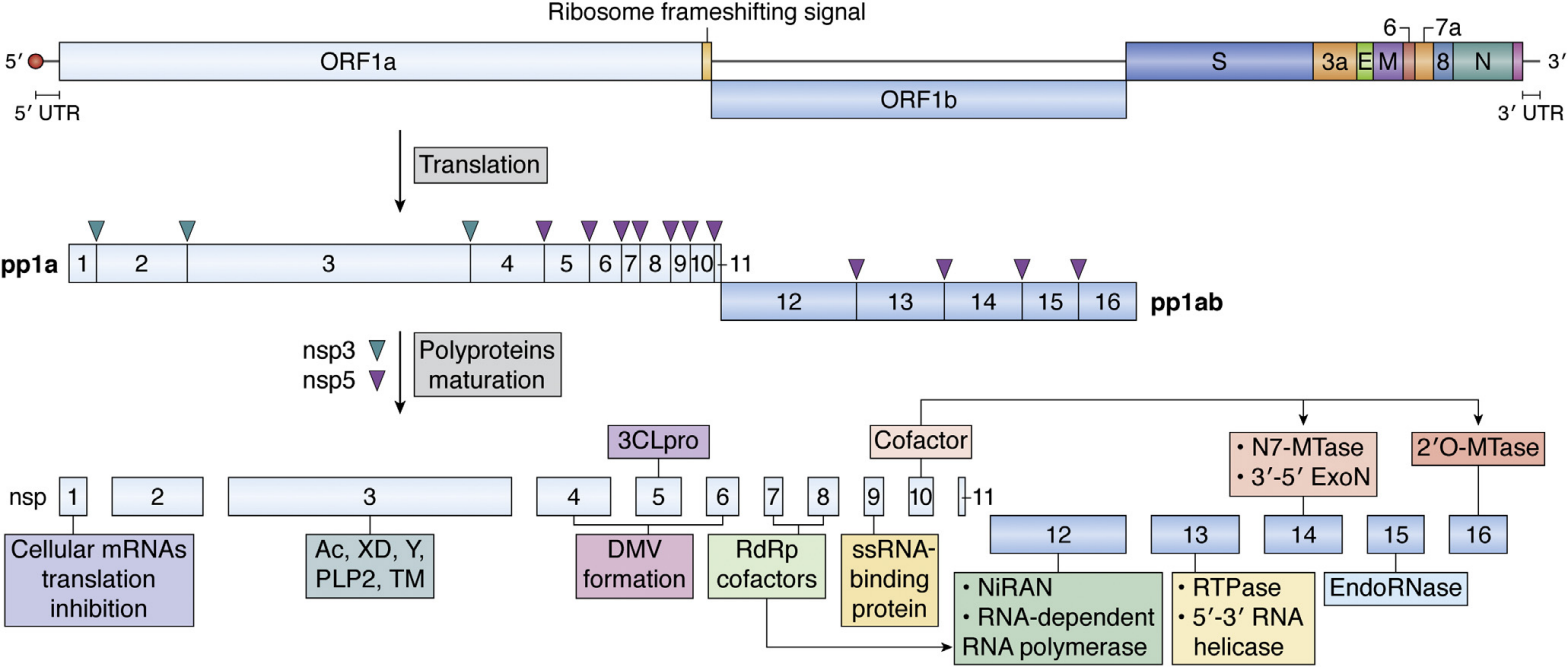
**SARS-CoV genome organization and nonstructural protein functions.** ORFs of the RNA genome are indicated as *boxes*, as well as the two untranslated regions (UTR) flanking the ORFs. Nsp1 to nsp16, coming from the proteolytic processing of the two polyproteins (pp1a and pp1ab) are shown. Confirmed functional domains are indicated above or below the nsps. 3CLpro, main protease; Ac, acidic domain; DMV, double-membrane vesicle; EndoRNase, endoribonuclease; ExoN, exoribonuclease; MTase, methyltransferase; NiRAN, nidovirus RdRp-associated nucleotidyltransferase domain; nsp, nonstructural protein; PLP2, papain-like protease 2; RdRp, RNA-dependent RNA polymerase; RTPase, 5′ RNA triphosphatase; TM, transmembrane domain; XD, X domain.

Similarly to all mammal +RNA viruses, the replication of CoVs occurs entirely in the cytoplasm. The viral RNA genome expression starts with the cap-dependent translation of the only two ORFs (ORF1a and ORF1b) that are directly accessible to cellular ribosomes (Fig. 2). This leads to the production of two large replicative polyproteins, named pp1a and pp1ab of 486 kDa and 790 kDa, respectively, in the case of SARS-CoV. Remarkably, the expression of the pp1ab polyprotein from ORF1b requires a programmed −1 ribosomal frameshifting (PRF) just upstream of the ORF1a translation termination codon, allowing it to bypass it and extending the pp1a in pp1ab (Fig. 2). While the *Nidovirales* frameshift event frequency at the ORF1a/1b junction is between 15% and 70%, the accepted efficiency for CoVs is around 50% (4, 17, 18, 19). Thereby, this frameshifting efficiency results in a two-fold overexpression of ORF1a-encoded proteins relative to ORF1b-encoded proteins. This unusually high PRF efficacy can be explained by the fact that ORF1b encodes five nsps, all harboring enzymatic activities (*e.g.*, the RdRp and the helicase) essential for viral replication (Fig. 2). The fine balance between ORF1a expression *versus* ORF1b is essential for the production of new viral particles. For instance, the SARS-CoV PRF alteration leads to a strong reduction in infectivity (20, 21).

After the production of the pp1a and pp1ab polyproteins, they are processed by two or three viral proteases, depending on the virus strain. In any case, the universal involvement of the ORF1a-encoded main protease (M^pro^), also known as 3CL^pro^ (due to its homology to the 3C^pro^ of picornavirus), is established (22). Remarkably, and in contrast to most CoVs, which use three protease activities for replicase polyprotein processing (23), the three CoVs causing major outbreaks of severe respiratory infections in humans encode only two of them (the papain-like protease in nsp3 and the 3CL^pro^ in nsp5) (Fig. 2). Thus, viral proteases allow the release of 16 mature nsps: pp1a yields nsp1 to nsp11, whereas pp1ab is cleaved into nsp1 to nsp10 and nsp12 to nsp16 (Fig. 2). The full polyprotein processing stage is essential for the viability of CoVs. Thus, with murine hepatitis virus (MHV), a commonly used CoV model, it has been shown that when the 3CL protease cleavage recognition site on polyproteins is mutated, no virus particles can be recovered (24). While the critical role of polyprotein pp1a processing has also been reported for arteriviruses, this family of viruses additionally expresses a wide variety of processing intermediate products from the C-terminal half of pp1a (25). They all contain nsp7, which is unique to arteriviruses but has an unknown function. By analogy to CoVs, these long-lived nsp7-containing intermediates may play different roles in viral RNA synthesis.

The 16 released functional CoV nsps, with a multitude of host proteins, form protein complexes and as such are referred to replication–transcription complexes (RTCs) (26, 27). Remarkably for CoVs, a viral structural protein, the nucleocapsid (N) protein, which is primary involved in the genome encapsidation, was shown to be required for viral replication and transcription (27, 28). This feature is unique for +RNA viruses and is reminiscent of negative-stranded RNA viruses where the N acts as a cofactor for the viral RdRp (29).

RTCs engage in the replication of new genomic RNAs as well as in the transcription of sg mRNAs encoding the four CoV structural proteins required for virion assembly and egress in addition to accessory proteins. The CoV RNA synthesis catalytic core is at least composed of the enzymes contained in nsp12, nsp13, nsp14, and nsp16, in association with their cofactors (nsp7 to nsp10) (Fig. 2). Other nsps confer advantages to the virus, such as nsp1, which mediates the shutdown of host mRNA translation (30, 31, 32), or nsp15, involved in innate immunity evasion (33, 34) (Fig. 2). All of the known functions for these nsps are presented in Figure 2. It is highly likely that “different” RTCs exist/coexist, each of them specialized in one function in order to maintain the optimal balance between the synthesis of new genomic RNA (replication) *versus* the different sg mRNAs (transcription). Indeed, as will be illustrated in this review, the CoV replicative cycle is fine-tuned by the dynamic associations and dissociations of, at least, some small ORF1a-encoded proteins (nsp7 to nsp10) (Fig. 2).

As with all eukaryote +RNA viruses, these viral replicase protein complexes are localized in specific organelles that are viral-induced from host cell membranes. There is a dual benefit for +RNA viruses, by concentrating all the elements for RNA genome replication as well as hiding viral negative-strand RNA and dsRNA intermediates from innate immunity receptor detection (for a review of this, see (35)). For CoVs, RTCs are located in virus-induced cytosolic double-membrane vesicles derived from endoplasmic reticulum membranes. The transmembrane domain of nsp3 is a major constituent of this viral organelle and certainly with the two other viral transmembrane proteins, nsp4 and nsp6, act as an assembly platform in anchoring RTCs in double-membrane vesicles (36).

## Discontinuous RNA synthesis for CoV structural and accessory gene expression

In addition to new viral RNA genomes, this tailored microenvironment will also produce sg mRNAs, which predominantly encode structural proteins, including spike (S), envelope (E), membrane (M), and nucleocapsid (N) proteins, as well some species-specific accessory proteins that are not essential for virus replication but are involved in pathogenesis (*e.g.*, modulation of the cellular innate immune response) (37). Indeed, ORFs encoding accessory proteins differ widely between various CoV lineages. For instance, while SARS-CoV contains eight accessory genes, SARS-CoV-2 has only six (38). Notably, the nested set of transcripts have the same 5′- and 3′-terminal sequence derived from the viral genome. Indeed, at the 5′ end, all sg mRNAs contain the common 5′ “leader” sequence derived from the 5′ end of the genome, followed by a “transcription regulating sequence” (TRS). Importantly, it was recently shown in the case of SARS-CoV-2 that the common 5′ leader sequence (and more precisely the first stem loop) protects viral mRNAs from nsp1-mediated translational inhibition (39). Thus, in addition to being essential for viral RNA synthesis, the 5′ leader sequence protects viral mRNAs from nsp1 action, as this viral protein-mediated deadlock seems to be a general feature in CoVs (30, 31, 32, 40).

**Figure 3:**
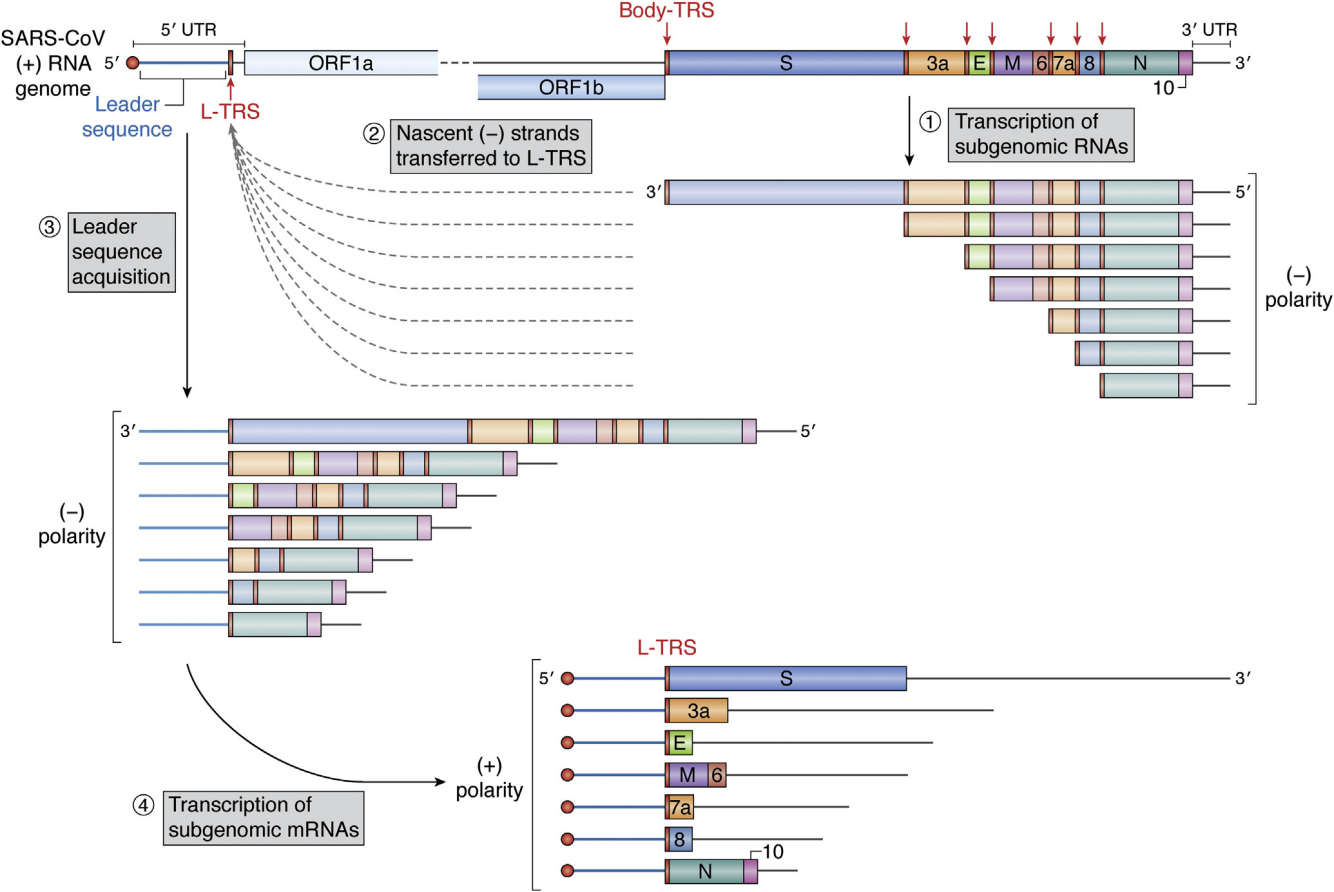
**The prevailing model for subgenomic messenger RNAs synthesis.***Step 1*, subgenomic RNA synthesis is initiated at the 3′ end of the genome and proceeds until it encounters one of the body-transcriptional regulatory sequence (body-TRS; in *red*), localized upstream of structural and accessory ORFs. *Step 2*, coronavirus genome RNA all contain a common 5′ Leader sequence fused to a TRS (named L-TRS). The Leader sequence may be added by a discontinuous synthesis of minus-sense subgenomic RNAs using genome RNA as a template. Through base-pairing interactions, the nascent transcript is transferred to the 5′ complementary Leader-TRS (L-TRS; in *red*). *Step 3*, the polymerase complex adds the complementary Leader sequence. *Step 4*, the negative-stranded subgenomic RNAs then serve as templates to continue viral sg mRNA production.

The different sg mRNAs are generated through a unique discontinuous transcription mechanism controlled by body-TRS. All TRSs contain a conserved 6 to 7 nt core sequence surrounded by variable sequences. Thus, a body-TRS is found just upstream of each ORF that encodes structural and accessory proteins, as well at the 5′ end of the genome (so-called leader-TRS) (Fig. 3). The prevailing model for sg mRNA transcription proposes that the leader sequence acquisition at the 5′ end of all sg mRNAs and whose template sequence is localized, some 20,000 nt upstream would occur during negative-strand synthesis (for a review of this, see (37, 41)). Briefly, during negative-strand synthesis, the RTC pauses when it crosses a body-TRS (Fig. 3; step 1) and switches from the 3′ portion of the genome to the 5′ leader-TRS (localized at the 5′ end of the genome) (Fig. 3; step 2). The template-switching event is thought to involve the stalling of the RdRp and then base-pairing between the 3′ end of the nascent transcript (by the complementary body-TRS sequence) and the leader-TRS near the 5′ leader sequence of the genome. It results in discontinuous transcription and in the fusion of a body-TRS to the leader sequence (Fig. 3; step 3). From the fused negative-strand intermediates, positive-strand mRNAs are then continuously transcribed (Fig. 3; step 4). Consequently, the RTC faces a decision problem when reaching a body-TRS: either to stop transcription and switch to the leader-TRS to produce a short sg RNA or to continue transcription and go through this body-TRS to another body-TRS and generate a longer sg RNA or even the genomic RNA. A major breakthrough occurred in 2014 on the mechanism ensuring the appropriate balance between the synthesis of different viral RNAs during a CoV’s life cycle (42). “Shorter” mRNAs are found in greater abundance, with, for instance, the sg mRNA encoding the N protein, which is the most abundant protein (Fig. 3). However, to survive, the virus has to pass the first body-TRS in an appropriate proportion in order to produce sufficient longer sg mRNAs and genomic RNA. Thereby, at certain stages of the replicative cycle, the N protein is phosphorylated by the host glycogen synthase kinase-3. The phosphorylated-N form allows for interaction with the cellular RNA helicase DDX1. This phosphorylated-N form–DDX1 complex increases the readthrough of the body-TRS and induces the synthesis of long sg mRNAs as well as genomic RNA (42). Given the abundance of S and M proteins, whose genes are furthest from the genomic 3′ end, it appears that this regulation mechanism is very effective.

Overall, the viral RTCs recognize different RNA promoter sequences and produce many viral RNAs. Indeed, CoVs produce the (+) RNA genome, a variable set of sg mRNAs (eight for SARS-CoV), and negative-sense RNA intermediates, being at least 20 different RNAs. In addition, numerous other viral RNAs coming from noncanonical discontinuous transcription events have been detected in the case of SARS-CoV-2 (15) as well for MHV (43), HCoV-229E (44), and toroviruses (45), but with currently unknown functions.

As outlined earlier, CoVs employ a unique, sophisticated multi-subunit machinery in order to replicate and maintain the integrity of their genome as well as transcribe a set of sg mRNAs encoding their structural and accessory proteins. Since 2010, the understanding of this machinery at the molecular level has significantly progressed. Most of the functional and structural information of the replicase machinery is available for SARS-CoV, MERS-CoV, and SARS-CoV-2, which we describe in the following sections.

## Identification of a highly active and processive RNA polymerase in CoVs

**Figure 4:**
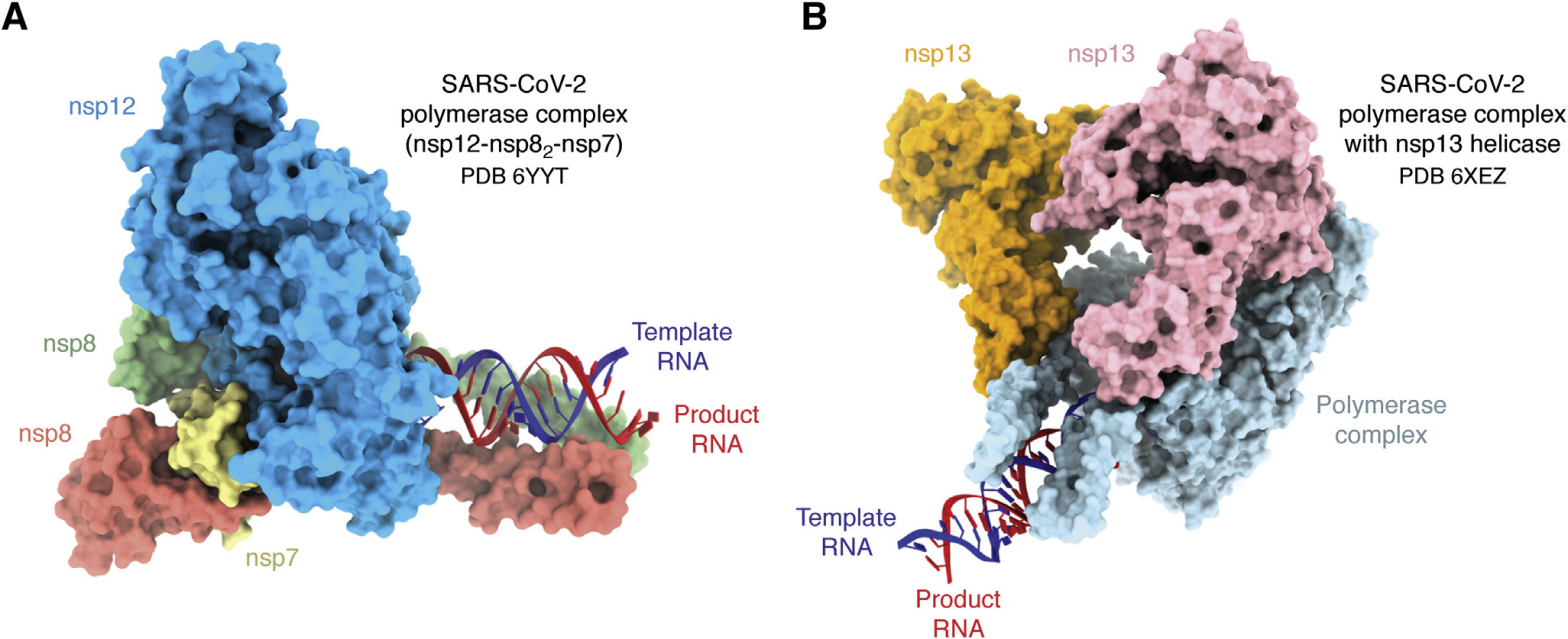
**Structural snapshots of the CoV-RNA–synthesizing machinery.***A*, SARS-CoV-2 polymerase complex (formed of nsp12-nsp8_2_-nsp7), in a complex with an RNA primer/template (57). *B*, SARS-CoV-2 polymerase complex in complex with two molecules of nsp13 helicase (62). Nsp13 in front of the polymerase complex may drive polymerase complex backtracking. This figure was created with ChimeraX (132). CoV, coronavirus; SARS-CoV-2, severe acute respiratory syndrome-CoV-2.

While the first CoVs that infect humans (HCoV-229E and HCoV-OC43) were isolated in the 1960s, it was only in 2014 that a highly active CoV RNA polymerase has been identified (46). Indeed, until then, only low *in vitro* nsp12-RdRp activity was obtained (47, 48). This discovery was guided by the size of the CoV genomes and the parallel made with DNA-dependent DNA polymerases that employ a processivity factor (*e.g.*, PCNA or β-clamp). Thereby, as putative cofactor(s), SARS-CoV nsp12 protein partners, previously identified by different methods (49, 50, 51), were added to nsp12-RdRp and the resulting RNA polymerase activity was measured. In the case of nsp8 as a potential RNA polymerase cofactor, nsp7 was concomitantly added as it was shown to stabilize nsp8 (52). Following this cofactor screening, strong nsp12 primer-extension activity as well as *de novo* RNA synthesis activity (*i.e.*, without any primer) in the presence of nsp8 and nsp7 was discovered (46). Then, optimization assays showed that nsp12-RNA polymerase activity was increased by the addition of a preformed nsp7–nsp8 complex (*via* a flexible peptide linker between both), suggesting a rate-limiting association between both. Further analyses showed that the nsp7–nsp8 complex activates and confers processivity to the nsp12-RdRp (46). Indeed, these two proteins are essential for nsp12-RNA–binding capacity, acting in a similar fashion to a clamp. Nsp7/nsp8 prevent the nsp12 falling off the RNA template during RNA polymerization, which is critical for efficient and fast viral replication (46). Importantly, the polymerase complex (formed of nsp12/nsp8/nsp7) is able to associate with an active bifunctional nsp14 (*i.e.*, ExoN and N7-methyltransferase (MTase) activities), hence retaining all associated enzymatic activities (46). This property is fundamental to go toward *in vitro* proofreading system reconstitution (discussed later). The first demonstration of nsp12-RdRp processivity factor requirements was performed for SARS-CoV (46) but has since been extended to MERS-CoV (53) and SARS-CoV-2 (54). Moreover, since 2019, the molecular details of the cooperation between nsp12 and nsp7/nsp8 have been obtained by cryo-EM 3D structure determination for SARS-CoV (55) and then for SARS-CoV-2 (56, 57, 58, 59, 60, 61, 62, 63, 64, 65, 66). Interestingly, the CoV nsp12 structure was only determined in the presence of both cofactors nsp7 and nsp8. As with all RdRps, the C-terminal nsp12 RdRp domain resembles a right hand, comprising the fingers, palm, and thumb subdomains. In all the available structures of the nsp12–nsp8–nsp7 complex, one nsp12 molecule interacts with one nsp7 and two molecules of nsp8 (Fig. 4*A*). An nsp7-nsp8 heterodimer binds to the thumb, and an additional copy of nsp8 binds to the finger domain. In light of these structural data, the nsp7-nsp8 heterodimer plays a critical role in stabilizing the polymerase domain, thereby enabling template recognition and binding (55). Moreover, in the presence of an RNA duplex (corresponding to a template product), the long α-helical amino-terminal extensions of the two nsp8 are stabilized and bound at the opposite sides of the polymerase active site cleft, forming “sliding poles” in which positively charged residues interact with and guide the exiting RNA (Fig. 4*A*) (57). Thus, these highly conserved helical extensions act as an electrostatic guide through which the product/template RNA duplex is extruded. This “sliding clamp” helps the polymerase to grip the RNA and prevent the premature dissociation of the RdRp from RNA during replication, thereby promoting polymerization processivity.

In contrast and surprisingly, biochemical RNA polymerase activity for arteriviruses, the short nidoviruses, remains elusive, and this is despite attempts to supplement arterivirus RdRp assays with the corresponding region of pp1a as a potential cofactor by analogy of the distantly related CoV RdRp cofactors (67).

## The nsp12 C-terminal RdRp domain

As outlined earlier, all characterized RdRps display a similar 3D fold, which resembles the shape of a right hand with thumb, palm, and fingers subdomains. For CoVs, RdRps exhibit a remarkable additional feature, with a long finger extension that intersects with the thumb subdomain to form a closed-ring structure and guide the RNA template entrance to the catalytic pocket (59). Additionally, the same catalytic mechanism, including seven critical catalytic motifs (A–G), is also shared between all RdRps (68). Motifs A to F are highly conserved for all viral RdRps, with motifs A and C, localized in the palm, involved in NTP binding and catalysis, respectively. Motif G is only found in certain RdRps, as a hallmark of primer-dependent RdRp, and interacts with the primer strand to initiate RNA synthesis. Indeed, RdRps can initiate RNA synthesis *via* two distinct mechanisms: either *de novo* or, in contrast, *via* a short RNA primer. For the latter, viruses have evolved different priming mechanisms, such as the formation of a covalent complex between the RNA primer and a viral protein (*e.g.*, poliovirus (69)) or cap-bearing 5′ fragments cleaved from cellular mRNAs (*e.g.*, influenza virus (70)). In the case of CoV RdRps, motif G has been identified (71), but the mechanism that provides an RNA primer to CoV RdRps is not yet known. A particularity of motif F of CoV RdRps is that it is comprised of a conserved β-hairpin loop (62). Thus, motif F directs the template RNA to the top, whereas underneath the motif is a channel that appears to be able to accommodate ssRNA. *Via* structural similarities to DNA-dependent RNA polymerases, this structural element was proposed to act as “strand-separating structural element” by dividing the active site cleft into two compartments.

In line with their exceptional genome size, CoV RNA-synthesizing machinery is the fastest known RdRp but also the least accurate (72, 73). This is not surprising given that there is growing evidence suggesting that intrinsic RdRp fidelity and speed have an inverse relationship (74, 75). Thus, CoV RdRp cooperates with the nsp14-ExoN to optimize both replication kinetics and fidelity (discussed below). Interestingly, two residues in MHV nsp12-RdRp were identified as determinants of RNA synthesis fidelity (76). Indeed, their mutation increases fidelity but only when nsp14-ExoN is inactive. Moreover, the intrinsic RdRp-increased fidelity does not completely compensate for the impaired fidelity associated with inactive nsp14-ExoN.

## The CoV nsp12 N-terminal extension domain

As with the viral genome, the size of the protein (nsp12) harboring the RdRp domain in CoVs is much larger than that of other (+) RNA viruses, with a size of ∼106 kDa against ∼65 kDa, traditionally. This feature comes from a unique amino-terminal extension that is inextricably linked to viral RNA polymerase activity and supports the latter *via* complementary enzymatic activities (see below). More broadly, all nidoviruses harbor this unique amino-terminal domain extension adjacent to the RdRp domain (9). From a structural point of view, the amino-terminal domain contacts the fingers and palm domains of the C-terminal RdRp domain. The first catalytic activity demonstration of this amino-terminal extension was done for the equine arteritis virus (EAV), belonging to *Arteriviridae* family. Self-nucleoside monophosphatylation of EAV nsp9 (ortholog of CoV nsp12) has been reported *in vitro*, with UTP and GTP as preferred substrates. Consequently, it was named NiRAN for nidovirus RdRp-associated nucleotidyltransferase domain (77). This nucleotidyl activity is dependent on Mn^2+^ and involves three conserved motifs: A_N_, B_N_, and C_N_ (where N stands for NiRAN). They contain a very small number of conserved residues among all monophyletic viral families but highly diverged *Nidovirales* order. Moreover, these conserved residues are essential for EAV in addition to SARS-CoV replication in cell culture (77). Further investigation has shown that the CoV nsp12 NiRAN domain exhibits structural similarity with the pseudokinase seleprotein-O, which transfers AMP from ATP to the Ser, Thr, and Tyr residues of protein substrates, activity termed AMPylation (78). In addition, another bacteria-selepotein-O-like protein, YdiU, was shown to catalyze the covalent attachment of UMP to the Tyr and His residues of diverse protein substrates (79). In this line, HCoV-229E and SARS-CoV-2 nsp12 were shown to transfer a single NMP from the NiRAN domain to the cognate nsp9, with a slight preference for UMP (80). The identification of a first NiRAN substrate in SARS-CoV-2 nsp9 and the structural similarities with protein kinases tend to support the idea that there must certainly be other NiRAN protein substrates of both viral and cellular origin. The role of the NiRAN nucleotidylation activity is still unknown, especially as the function of nsp9 is also enigmatic with the exception of its RNA-binding property and essential role in viral replication (described later) (81). Nevertheless, it is tempting to speculate that it might be involved in the protein priming of RNA synthesis, as employed by *Picornaviridae* (69). Indeed, as already mentioned, bioinformatics analyses have identified motif G in the CoV nsp12-RdRp domain, conserved residues involved in RNA primer/template recognition (71).

Moreover, the NiRAN domain appears to possess more than one function. Indeed, very recently it has been shown that SARS-CoV-2 nsp12 NiRAN possesses guanylyltransferase (GTase) activity, the first step for the formation of the cap structure at the 5′ end of viral mRNAs (63). Universally, the cap structure plays essential roles in the living world by promoting the initiation of protein translation, protecting mRNAs, and helping, in the case of RNA viruses, to escape host immune recognition (82). The cap structure formation in CoVs consists of four sequential enzymatic reactions: (i) a 5′ RNA triphosphatase that removes the γ-phosphate at the 5′-triphosphate end of the mRNA, (ii) a GTase that transfers a GMP to the mRNA 5′-diphosphate end, (iii) an N7-MTase that methylates the cap guanine at the N7-position, producing the cap-0 structure, and finally (iv) a 2′O-MTase that methylates the ribose in the 2′-O-position of the first transcribed nucleotide, forming the cap-1 structure. RNA triphosphatase activity was identified in nsp13, which also harbors RNA helicase activity (discussed later) (83). N7- and 2′O-MTases activities are carried out by nsp14 and nsp10/nsp16, respectively (84, 85, 86). Of note, the cap formation pathway of CoV mRNAs is highly regulated by nsp10, which is essential for nsp16 to 2′O methylation activity (87), and, conversely, nsp9 through its N-terminus end represses SARS-CoV-2 NiRAN GTase activity (63).

## Nsp7 and nsp8: The nsp12-RdRp cofactors

### Nsp8

Nsp8 is unique and well-conserved protein to CoVs. Conflicting data exist regarding enzymatic activity carried out by nsp8, which may come from experimental design as well as the CoV strain used. In 2006, SARS-CoV nsp8 was shown as a mandatory *de novo* initiating RNA polymerase, able to synthesis products of less than six nucleotides (88). This second noncanonical RdRp was proposed to act as a primase in providing primers to the main nsp12-RdRp. Indeed, as previously mentioned, CoV RdRps harbor motif G, which is implicated in primer/template recognition (71). Therefore, it has been proposed that nsp12 initiates RNA synthesis through primers produced by nsp8. Nsp8 *de novo* polymerase activity has also been demonstrated for two other CoVs (FCoV and HCoV-229E) (89). In contrast, in 2012, another study showed that SARS-CoV nsp8 (alone or in a complex with nsp7) is able to extend primed RNA templates (90). Lastly, a subsequent study on HCoV-229E nsp8 only demonstrated adenylyltransferase activity and therefore proposed a role in viral RNA poly(A) tail synthesis (91). To conclude, nsp8-primase activity still remains an open question.

Regarding nsp8 structural studies, to date, three crystal structures of nsp8 have been reported, for SARS-CoV (52), feline CoV (89), and SARS-CoV-2 (92), and each time in the presence of nsp7, which is essential to stabilize nsp8. The superimposition of the different CoV nsp8 monomers reveals that the fold is quite conserved between the different structures, with the characteristic “golf-club” nsp8 shape. However, nsp7–nsp8 complex stoichiometries are quite different depending on the CoV and suggest a remarkable architectural plasticity to accommodate multiple functions (93). The SARS-CoV nsp7/nsp8 structure assembles in an hexadecamer (*i.e.*, eight molecules of nsp7 with eight molecules of nsp8), forming a ring with a central channel that through its size and electrostatic properties can perfectly accommodate a dsRNA (52). The crystal structure of the feline CoV nsp7–nsp8 complex forms a heterotrimer *via* the association of two nsp7 molecules with one molecule of nsp8, and without the formation of a hollow structure (89). Lastly, in the case of SARS-CoV-2, a dimer of nsp7/nsp8 dimers was crystallized, as well as without any channels (92). Thereby, these different assembly states of nsp7 and nsp8 may act as molecular switches of viral replication in function of the viral infection stage.

As mentioned above, while it is undeniably accepted that nsp8 is the processivity factor for nsp12-RNA polymerase activity, a second noncanonical RNA polymerase activity remains controversial. All nsp12/nsp8/nsp7 available structures assemble with a [1:2:1] stoichiometry, and the positions of the two nsp8 molecules are inconsistent with the formation of a primase active site in nsp8 (55, 56, 57, 58, 59, 60). Nevertheless, we must keep in mind that structures correspond to snapshots of a multitude of possible conformations during the viral cycle.

In addition to its essential role in viral RNA synthesis, it has recently been shown that the SARS-CoV-2 nsp8 disrupts protein trafficking to the cell membrane upon infection, contributing to host defense suppression *via* the shutdown of the IFN response (39). Moreover, the IFN response suppression is potentiated by SARS-CoV-2 nsp1, nsp9, and nsp16. This host defense suppression seems to be more pronounced for SARS-CoV-2 than for SARS-CoV and MERS-CoV infections, whereas nsp1, nsp8, nsp9, and nsp16 are highly conserved in CoVs, most certainly involving other viral determinants.

### Nsp7

The precise role of nsp7 as a cofactor for nsp12-RdRp activity is less clear. For instance, an experimental approach has expressed in insect cells the [nsp5-nsp7-nsp8-nsp12] cassette where the nsp5-protease activity allows for the release of the four proteins and thus enables them to mimic the natural assembly of the nsp12–nsp8–nsp7 replication complex. This approach was employed for SARS-CoV, MERS-CoV, and SARS-CoV-2, and in each case, an active RNA polymerase complex was recovered but made up of only nsp12 and nsp8 (53, 54). In addition, whereas SARS-CoV nsp7 mutations *in vitro* significantly affect nsp12-RdRp activity, they have a limited effect *in vivo*, only slightly reducing the viral replication efficiency (46). Nevertheless, and interestingly, a very recent study analyzed a large cohort of sera from COVID-19-infected patients and defined their antibody response profiles against the SARS-CoV-2 proteome. They showed that in addition to S and N proteins, some nonstructural and accessory proteins also elicit prevalent antibody responses (94). In addition, the intensity of the humoral response against nonstructural and accessory proteins is correlated with disease severity. Among them, strong IgG responses are detected against nsp8, nsp12, and nsp7. While a high correlation of IgG responses was found between nsp12 and nsp8, the correlation between nsp7 and nsp8 was less significant. Based on structural data of the polymerase complex, most of the nsp7 surface is rather inaccessible in contrast to nsp8 and nsp12, limiting the exposure of antigenic sites. Therefore, the high antibody rate elicited by nsp7 suggests that this protein may exist in other forms rather than in a complex with nsp8 and nsp12, thus having other biological functions still to be discovered.

## CoV nsp9, a key player in the replication/transcription catalytic core

As illustrated with nsp7, nsp8, and nsp10, CoVs have acquired unique, well-conserved, and small ORF1a-encoded proteins to regulate and orchestrate their replicative cycle. For all of them, neither bioinformatics analyses nor isolated structure function data of these proteins had made it possible to anticipate such dynamic regulation mechanisms. Nevertheless, these studies are essential to pave the way toward a comprehensive understanding of this multi-subunit RNA synthesis machinery. In the case of CoV nsp9, a protein of about 13 kDa, its precise functions are still elusive and only fragmentary information is available, likely highlighting pleiotropic effects. Nsp9 can bind ssRNA or DNA (81, 95, 96). Moreover, nsp9 was shown to interact with the viral-RdRp (nsp12) and colocalize with nsp7-nsp8-nsp10 in mouse CoV MHV (49, 50, 51). By reverse genetics analysis, still in MHV, nsp9 (and nsp8) were shown to specifically and critically interact with a conserved *cis*-acting RNA element near the 3′ end of the CoV genome (97). The recent discovery of nsp9-UMPylation by nsp12-NiRAN makes it tempting to propose that nsp9 could be involved in the initiation of minus-stranded RNA synthesis *via* serving as a primer by complementarity with the genomic poly(A) tail (80). Whether nsp9 is active as a monomer or a dimer remains to be determined (63, 66, 96). Indeed, the crystal structure of the SARS-CoV nsp9 revealed a dimeric assembly, and mutations of conserved residues at the dimerization interface (G100, G104) impaired viral proliferation (96). However, the recent cryo-EM 3D structure of the nsp12–nsp8–nsp7–nsp13–nsp9 complex shows one nsp9 molecule tightly bound to nsp12 (63). Notably, nsp9 G100 and G104, as well as other residues mediated nsp9 dimerization, face nsp12, and are part of the nsp9–nsp12 interface. Interestingly, and as outlined earlier, nsp9 as a monomer deeply inserts its N-terminus into the catalytic center of nsp12-NiRAN and bonds with the GDP moiety (63), which thereby inhibits NiRAN GTase activity (63). Among the multitude of RNAs produced by CoVs, only viral messenger RNAs need to and must be capped. The inhibition of the first step of cap formation by nsp9 could avoid the “dispersion” of cellular ribosomes toward nonsense proteins.

## CoV RNA proofreading mechanism through nsp14-ExoN activity

CoV ExoN motifs in the N-terminal domain of nsp14 were identified in 2003 during the full analysis of the SARS-CoV genome, which was just emerging. It was therefore classified in the superfamily of DEDD exonucleases, where this name derives from the four conserved active site residues of the three canonical motifs (4). This catalytic site corresponds to a 3′-5′ ExoN, which also includes the proofreading domains of many DNA polymerases (98). In 2006, *in vitro* nsp14-ExoN enzymatic activity was demonstrated (99). Since then, there has been more and more evidence that the poor fidelity of CoV RNA polymerases is enhanced by the cognate viral-encoded 3′-to-5′ ExoN, which furthermore may have promoted the expansion of large nidoviral genomes to their current size (4, 8). Indeed, ExoN activity is absent in smaller (<15 kb) nidovirus genomes (*e.g.*, arteriviruses) (Fig. 1). One major breakthrough in the demonstration of CoVs’ proofreading mechanism comes from reverse genetic studies, first on MHV in 2007 (100) and then on SARS-CoV in 2010 (14). Thus, in infected cell cultures, nsp14-ExoN activity invalidation is maintained stably for more than ten passages and leads to 15- to 20-fold increased mutation frequencies across viral progeny genomes compared to WT viruses (14, 100). With this approach, the SARS-CoV mutation rate has been estimated to 2 × 10^−6^ mutations per site per round of replication, whereas for all other RNA viruses, error rates instead range from 10^−3^ to 10^−5^ mutations per site per round of replication (14). Interestingly, CoVs lacking ExoN activity are more susceptible to nucleoside analogs than WT viruses (101, 102). Lastly, a mouse-adapted SARS-CoV ExoN inactivation leads to stable attenuated virus virulence in animals (103). This strategy could be used for the rational design of live, attenuated CoV vaccines. Unfortunately, and in contrast to MHV and SARS-CoV, nsp14 ExoN KO mutants led to a nonviable phenotype for HCoV-229E (99), TGEV (104), MERS-CoV, and SARS-CoV-2 (105, 106), preventing *in vivo* studies for these CoVs and the development of a vaccine approach. Thus, the ExoN activity for at least these four CoVs seems to possess an additional function at early stages of replication.

Fortunately, structural and biochemical studies have advanced our understanding of the role of nsp14-ExoN in error correction. In 2012, using recombinant proteins, it was shown that SARS-CoV nsp14-ExoN activity was stimulated by nsp10 in a dose-dependent manner, up to a 35-fold stimulation reached with a fourfold excess of nsp10 over nsp14 (107). The crystal structure of the SARS-CoV nsp10–nsp14 complex revealed the molecular mechanism by which nsp10 stimulates nsp14-ExoN activity only (73). Nsp10 settles in a cavity, thereby stabilizing the active site of the nsp14-exonuclease domain. Thus, nsp10 acts as a “scaffolding” protein, ensuring the proper conformation of the ExoN active site, thereby enhancing substrate hydrolysis. Then, it was shown that the SARS-CoV nsp10–nsp14 complex solely hydrolyzes dsRNA in a 3′ to 5′ direction. The nsp10-nsp14-ExoN complex is also able to excise a single 3′-end mismatched nucleotide, mimicking an erroneous nsp12-RdRp incorporation, regardless of the nature of the single mismatch (107). Interestingly, the same study indicates that a mismatched 3′-end base pair might be preferred to a Watson–Crick substrate. Nevertheless, nsp10/nsp14-ExoN excision capability is strongly decreased with two, three, or four mismatched nucleotides introduced at the 3′ end and totally inactive on dsRNA substrates bearing 3′-end ribose modifications (such as puromycin, phosphate, or 2′,3′-cyclic phosphate generated by nsp15-NendoU cleavage) (107).

Through the different achievements previously described, the *in vitro* reconstitution of the SARS-CoV proofreading machine came in 2018 (46, 49, 88, 107). Thereby, after the addition of the nsp10-nsp14–ExoN complex, the SARS-CoV polymerase complex has been able to resume polymerase synthesis from a primer/template bearing an A:A mismatch. Indeed, these non-Watson–Crick base pairs are poorly extended by the polymerase complex. The resulting RNA products were sequenced, and ∼90% of the sequenced clones were repaired (*i.e.*, U instead of A nucleobases were found). Through this pathway, the antiviral compound ribavirin 5′-monophosphate was significantly incorporated by the SARS-CoV polymerase complex but also readily excised from RNA. This result may explain its limited efficacy on CoV-infected patients (108, 109). Moreover, the rate of nsp10/nsp14-mediated ribavirin excision is about fourfold faster than that of an A:A mismatch (73). CoVs are the first RNA viruses known to use an RdRp processivity factor to expedite the replication of their ∼30-kb RNA genome as well as 3′-5′ ExoN activity to excise nucleotide misincorporation, both features fitting perfectly into a unique RNA proofreading system.

Concerning the structural data of nsp14, two crystal structures of full-length SARS-CoV nsp14 (73, 110) and two of SARS-CoV-2 nsp14 with only the ExoN domain (111, 112) have been solved, all in a complex with nsp10 as an ExoN cofactor. This structural information revealed that SARS-CoV and SARS-CoV-2 ExoN are in fact DEED exonucleases instead of DEDD, as originally predicted. Nevertheless, DEDD ExoNs are also found for different nidovirus taxa. Lastly, from these studies, a fifth catalytic residue (His) was revealed. Thus, five catalytic residues (DEEDh or DEDDh) coordinate 2 Mg^2+^ ions and assist in the removal of nucleotides.

As mentioned previously, nsp14 is a bifunctional protein with an N-terminal ExoN domain and a C-terminal N7-MTase domain, involved in viral mRNA capping. In the two SARS-CoV nsp10/nsp14 structures, these two domains are physically independent. However, mutagenesis analyses have shown that they are functionally intertwined, as N7-MTase activity depends on the integrity of the N-terminal ExoN domain (84, 110) and some residues in the MTase domain are important for ExoN activity (46, 113). Although SARS-CoV and SARS-CoV-2 nsp14 exhibit high sequence identity, the SARS-CoV-2 nsp14-ExoN domain retains ExoN enzymatic activity despite the absence of the nsp14-MTase domain (114). Indeed, the SARS-CoV-2 nsp14-ExoN domain conserved an integral ExoN fold, which is quite conserved with that of SARS-CoV. This result emphasizes once again the singular character of each CoV, even if high sequence conservations are observed.

In addition, in the case of SARS-CoV, structural analyses reveal a hinge connector domain between the two nsp14 catalytic domains, exhibiting a high flexibility with lateral and rotational movements. The nsp14 hinge region is, at least, essential for the interaction with the viral polymerase (73).

Based on these results, a key question arises regarding the implication of nsp10 in the nsp14-mediated proofreading mechanism. Indeed, strong nsp14-ExoN stimulation activity driven by nsp10 raises the issue of the preservation of viral dsRNA intermediates. In addition to the *Nidovirales* order, arenaviruses are the only other RNA viruses known to encode 3′-5′ ExoN activity. For the latter, it has been implicated in immune evasion by possibly degrading viral dsRNA (115). Given the high potency of nsp14-ExoN activity in the presence of nsp10, we favor the hypothesis that nsp10/nsp14-ExoN activity is rather involved in innate immune evasion (35, 104, 112, 116, 117). In particular, a very recent study on SARS-CoV-2 has shown that nsp14 shuts down host innate immune responses *via* translation inhibition, and this action requires ExoN and N7-MTase active sites. In addition, inhibition is increased by the formation of the nsp14–nsp10 complex (117). In the eventuality that nsp10 is not involved in the proofreading pathway, several nsp14 partners have already been identified in infected cells and so can act as an nsp14-ExoN activity stabilizing factor (118).

Finally, even if our knowledge on nsp14 has considerably progressed there are still many shadow areas, mainly concerning the dynamics, kinetics, and regulation of different enzymatic activities. For example, nsp14 comprises two antagonistic actions: 5′ end mRNA capping and 3′→5′ mismatch excision activity. The recent cryo-EM 3D structure of the nsp12–nsp7–nsp8–nsp9–nsp13–nsp10–14 complex (66), with a paired template–primer RNA, showed that the nsp14 ExoN domain only is involved in protein–protein interactions. This domain contacts nsp9 and nsp12-NiRAN while the nsp14-MTase domain points outward from the whole assembly. In this configuration, the path for transferring pre-mRNA with GpppA from the NiRAN catalytic center to the nsp14-MTase to generate the cap ^7Me^GpppA is not clearly identified. Moreover, the active site of nsp14-ExoN being far away from the exit of replicated RNA, proofreading is unlikely.

All in all, CoV nsp14 plays a central role in replication with pleiotropic actions, encompassing RNA proofreading, cap methylation, interference with host innate immune responses, and, as recently demonstrated, in RNA recombination (106). Although recombination events have long been proposed in CoVs (119), the involvement of nsp14 in this process has only very recently been identified through the advent of RNA next-generation sequencing technology. The depletion of nsp14-ExoN activity in MHV leads to an alteration in recombination patterns, corresponding to a decrease in sg mRNA populations and an increase in defective viral genomes (106).

## CoV nsp13 helicase: Essential for replication but with ill-defined functions

CoV helicase, similar to the 3CL-like protease and the NiRAN-RdRp domains, is one of the most evolutionarily conserved proteins in nidoviruses. CoV nsp13 is organized into three domains, which include a unique N-terminal Cys/His-rich domain with three zinc atoms (called the zinc-binding domain; ZBD), a beta-barrel domain, and a C-terminal superfamily 1 helicase core with two RecA-like subdomains (120, 121). The individual domains are closely related to the eukaryotic domains of Upf1 helicase, a key factor in the cellular nonsense-mediated mRNA decay process (122). This structural similarity leads to the hypothesis that either nidoviruses’ helicase may have a Upf1-like role in the posttranscriptional quality control of viral RNA synthesis and/or may interfere with the nonsense-mediated mRNA decay pathway to avoid viral RNA degradation (123). Nevertheless, the three nsp13 domains taken as a whole are unique to nidoviruses. Thereby, the ZBD interacts extensively with the helicase core and these relays of interactions are essential for CoV helicase activity (124). Biochemical characterizations have shown that CoV nsp13 exhibits multiple enzymatic activities, which include the hydrolysis of NTPs and dNTPs, unwinding of DNA and RNA duplexes with 5′-3′ polarity, and RNA 5′-triphosphatase activity (83, 125). Moreover, for SARS-CoV, it has been shown that nsp12, *via* direct protein–protein interaction, can enhance nsp13-helicase activity (121). Mutagenesis studies have demonstrated an essential role of nsp13 in CoV replication (126, 127). In addition, for avian infectious bronchitis virus, which is a gamma-CoV, a single mutation in nsp13 significantly attenuated the synthesis of subgenomic transcripts without affecting full-length genome production (128). The same phenotype was observed in arterivirus counterparts. Indeed, a mutation just downstream of the ZBD of the nsp10 helicase was shown to decrease arterivirus sg RNA transcription without affecting genomic replication (129). Nevertheless, the molecular contributions of CoV nsp13 to viral replication and transcription remain poorly understood.

On the same strand, CoV nsp13 helicase polarity (5′→3′) that runs opposite to that of the RNA polymerase (3′→5′) means that direct cooperativity between both, that is, helicase unwinding the RNA template for copying, cannot be considered. A solution to this conundrum comes from recent structural studies of *in vitro* SARS-CoV-2 replicative machinery reconstitutions. Indeed, in these structures, it was mostly revealed two nsp13 molecules sit above the polymerase complex (made up of nsp12-nsp8_2_-nsp7) (61, 62). These two nsp13 molecules have extensive interactions with the polymerase complex, while only one also interacts with the 5′ end overhang of a RNA template (Fig. 4*B*). By analogy to cellular DNA and RNA polymerases, it was proposed that the RNA-bound nsp13 molecule would push the RdRp backward on the template RNA. Indeed, this reversible phenomenon, called backtracking, is well-known and -characterized in eukaryotic and prokaryotic cells (130, 131). This process for CoV has recently been supported by the discovery that SARS-CoV-2 nsp13 stimulates the backtracking of the RdRp, allowing the 3′ end of the nascent RNA to extrude out of the RdRp (65). The availability of the 3′ end of the RNA product may be involved in granting ExoN access for proofreading or template-switching during subgenomic transcription. Indeed, RdRp backtracking may play a role in discontinuous RNA synthesis by exposing the complementary body-TRS sequence at the 3′ end of the nascent minus-strand sg RNA, thus mediating template-switching (Fig. 3). This role is supported by the avian infectious bronchitis virus reverse genetics experiments previously mentioned (128). To conclude, based on current knowledge, CoV nsp13 appears to act more as a translocase than a helicase, allowing for the fine-tuning of the regulation of, at least, proofreading and template-switching processes.

## Conclusion

Since the emergence of SARS-CoV in 2003, significant progress has been made in the characterization of key mechanisms and proteins involved in the replication of the RNA genome. The emergence of SARS-CoV-2 has literally boosted our knowledge, notably regarding the structural organization of the key viral proteins involved in these processes. Nevertheless, many gray areas remain such as the transcription mechanisms of the sg mRNAs with potential “strand jump”. In the future, with an *in vitro* CoV reconstituted replicative system, combined with advances in deep sequencing, CoVs’ transcriptome could be fully characterized and defined. The temporality and spatial arrangement of proofreading during RNA synthesis also remain to be unraveled.

Finally, it is important not to be limited to SARS-CoV-2/pathogen CoVs but to extend our knowledge to the different families of viruses forming this exceptional and fascinating *Nidovirales* order.

## Conflict of interest

The authors declare that they have no conflicts of interest with the contents of this article.
